# Regulatory mechanisms of microRNAs in endocrine disorders and their therapeutic potential

**DOI:** 10.3389/fgene.2023.1137017

**Published:** 2023-02-21

**Authors:** S. Janin Ledesma-Pacheco, Andrea G. Uriostegui-Pena, Estefania Rodriguez-Jacinto, Elizabeth Gomez-Hernandez, Carolina Estrada-Meza, Antara Banerjee, Surajit Pathak, Luis M. Ruiz-Manriquez, Asim K. Duttaroy, Sujay Paul

**Affiliations:** ^1^ Tecnologico de Monterrey, School of Engineering and Sciences, Queretaro, Mexico; ^2^ Chettinad Academy of Research and Education (CARE), Chettinad Hospital and Research Institute (CHRI), Department of Medical Biotechnology, Faculty of Allied Health Sciences, Chennai, India; ^3^ Tecnologico de Monterrey, Escuela de Medicina, Monterrey, Mexico; ^4^ Department of Nutrition, Institute of Basic Medical Sciences, Faculty of Medicine, University of Oslo, Oslo, Norway

**Keywords:** miRNAs, endocrine disorders, diabetes mellitus, thyroid diseases, osteoporosis, pituitary tumors

## Abstract

MicroRNAs (miRNAs) are small endogenous non-coding RNA molecules capable of regulating gene expression at the post-transcriptional level either by translational inhibition or mRNA degradation and have recently been importantly related to the diagnosis and prognosis of the most relevant endocrine disorders. The endocrine system comprises various highly vascularized ductless organs regulating metabolism, growth and development, and sexual function. Endocrine disorders constitute the fifth principal cause of death worldwide, and they are considered a significant public health problem due to their long-term effects and negative impact on the patient’s quality of life. Over the last few years, miRNAs have been discovered to regulate various biological processes associated with endocrine disorders, which could be advantageous in developing new diagnostic and therapeutic tools. The present review aims to provide an overview of the most recent and significant information regarding the regulatory mechanism of miRNAs during the development of the most relevant endocrine disorders, including diabetes mellitus, thyroid diseases, osteoporosis, pituitary tumors, Cushing’s syndrome, adrenal insufficiency and multiple endocrine neoplasia, and their potential implications as disease biomarkers.

## 1 Introduction

The main function of the endocrine system’s highly vascularized ductless organs is to produce hormones that can control metabolism, growth, development, and sexual function (Thi et al., 2017; Perle, 2021). The major glands that comprise the endocrine system are the adrenal, hypothalamus, pituitary, parathyroids, pineal body, thyroid, and ovaries or testes (Malespin & Nassri, 2019). The epithelial cells of the organs secrete their specific hormone product into the bloodstream, which binds to specific receptors in the target organs, affecting cellular functions (Perle, 2021). Endocrine disorders occur with the dysregulation of hormone levels or if the body does not respond to hormones appropriately (Lause et al., 2017) and constitute the fifth principal cause of death worldwide. Moreover, they are a significant public health issue since they can substantially decrease patients’ quality of life and cause long-term disabilities (Crafa et al., 2021).

MiRNAs are small (21–24 nucleotides long) endogenous non-coding RNA molecules that post-transcriptionally regulate gene expression either by degradation of mRNA or translational inhibition (Xiang et al., 2017; de Sousa et al., 2019). Biogenesis and processing of the miRNA molecules start in the cell’s nucleus, where RNA polymerase II transcribes the genes that codify the miRNA into the hairpin-structured primary miRNA, which is converted into a shorter stem-loop precursor miRNA by the microprocessor complex that consists of the enzymes Drosha and DGCR8. Afterward, the precursor miRNA is exported to the cytoplasm due to an interaction with Exportin 5, to be further processed into a mature miRNA/miRNA* duplex by the action of RNase III Dicer and trans-activation responsive RNA-binding protein. Next, a helicase separates the duplex so that the guide strand can be integrated into the RNA-induced silencing complex, also known as RISC, which is guided by the AGO2 protein. Finally, the RISC-miRNA complex identifies specific mRNA targets by base complementarity leading to translational inhibition or mRNA degradation (Paul et al., 2021; Ruiz-Manriquez et al., 2022).

Alternatively, numerous non-canonical miRNA biogenesis pathways have been identified and thoroughly revised (Treiber et al., 2018; Stavast and Erkeland, 2019). These pathways employ various combinations of the canonical pathway proteins, primarily Drosha, Dicer, exportin 5, and AGO2 (Kim et al., 2016; O’Brien et al., 2018). The non-canonical miRNA biogenesis can be divided into Dicer-independent and Drosha/DGCR8-independent pathways (Annese et al., 2020). Pre-miRNAs produced by the Drosha/DGCR8-independent pathway resemble Dicer substrates. Mirtrons, which are created during splicing from the introns of mRNA, is an illustration of such pre-miRNAs. The 7-methylguanosine (m7G)-capped pre-miRNA serves as another illustration. Without Drosha cleavage, these developing RNAs are exported directly to the cytoplasm by exportin 1. Conversely, Dicer-independent miRNAs are processed by Drosha from endogenous short hairpin RNA (shRNA) transcripts. These pre-miRNAs demand AGO2 terminate their maturation within the cytoplasm since they are of insufficient length to be Dicer-substrates.

According to most studies, miRNAs induce translational repression, mRNA deadenylation, and decapping by binding to a specific sequence at the 3′UTR of their target mRNAs (Dexheimer and Cochella, 2020). Nevertheless, miRNA binding sites have also been found in the 5′UTR, coding sequence, promoter regions, and other mRNA regions (Treiber et al., 2018). While the interaction of miRNAs with the promoter region has been reported to induce transcription, miRNA binding to the 5′UTR and coding regions has been shown to silence the expression of specific genes (O’Brien et al., 2018; Liu et al., 2021; Xiang et al., 2022). Interestingly, these molecules are found to regulate about 60% of genes of the human genome (Zhang and Wang, 2017), and currently, 1,917 precursors (hairpin portion of a miRNA transcript) and 2,654 mature human miRNA sequences are deposited to the microRNA database/miRbase (miRBase, 2022; Kozomara et al., 2019; Kozomara and Griffiths-Jones, 2014).

Over the past decades, it has been demonstrated that miRNAs can regulate hormone production, activity, and target cell responsiveness (Peng and Wang, 2018). For instance, miRNAs can directly target genes encoding hormones or enzymes involved in hormone production or metabolism, affecting hormone concentrations; and proteins that modulate hormone actions, such as antagonists, can also be regulated by miRNAs; also, many studies have demonstrated that hormones regulate miRNAs, and such regulation can occur at the level of transcription or processing resulting in a complex regulatory network that ultimately impacts cell homeostasis (Peng and Li, 2022). Moreover, miRNAs can target hormone receptors and intracellular signaling molecules to alter target cell responses (Derghal et al., 2016; Bayraktar et al., 2017). Recently, evidence postulated that miRNAs could be secreted to the extracellular milieu and act as endocrine factors, performing endocrine and paracrine crosstalk between cells and tissues (Wu et al., 2022).

Recently, miRNAs have been demonstrated to regulate various processes associated with endocrine disorders, such as high-glucose-induced apoptosis, insulin secretion, and proliferation (Wang et al., 2020), as well as glycolipid metabolism (Zhang et al., 2020) in diabetes mellitus; abnormal ciliogenesis (Martínez-Hernández et al., 2019) in thyroid diseases; osteoclast and osteoblast differentiation (Li et al., 2018; Wang et al., 2021) in osteoporosis; proliferation and apoptosis (He et al., 2018; He et al., 2020) in pituitary tumors; proliferation (Vetrivel et al., 2022) in Cushing’s syndrome; steroidogenesis (Bitetto et al., 2022) in adrenal insufficiency; and adrenocortical proliferation (Li et al., 2021) in multiple endocrine neoplasia; among others. The association of these small RNA molecules in endocrine disorders could be advantageous in developing novel diagnostic and therapeutic tools.

The present review aims to illuminate the most recent and relevant information about the potential role of microRNAs in endocrine disorders such as diabetes mellitus, thyroid diseases, osteoporosis, pituitary tumors, Cushing’s syndrome, adrenal insufficiency, multiple endocrine neoplasia, as well as their diagnostic and therapeutic potential.

### 1.1 Diabetes mellitus

**FIGURE 1:**
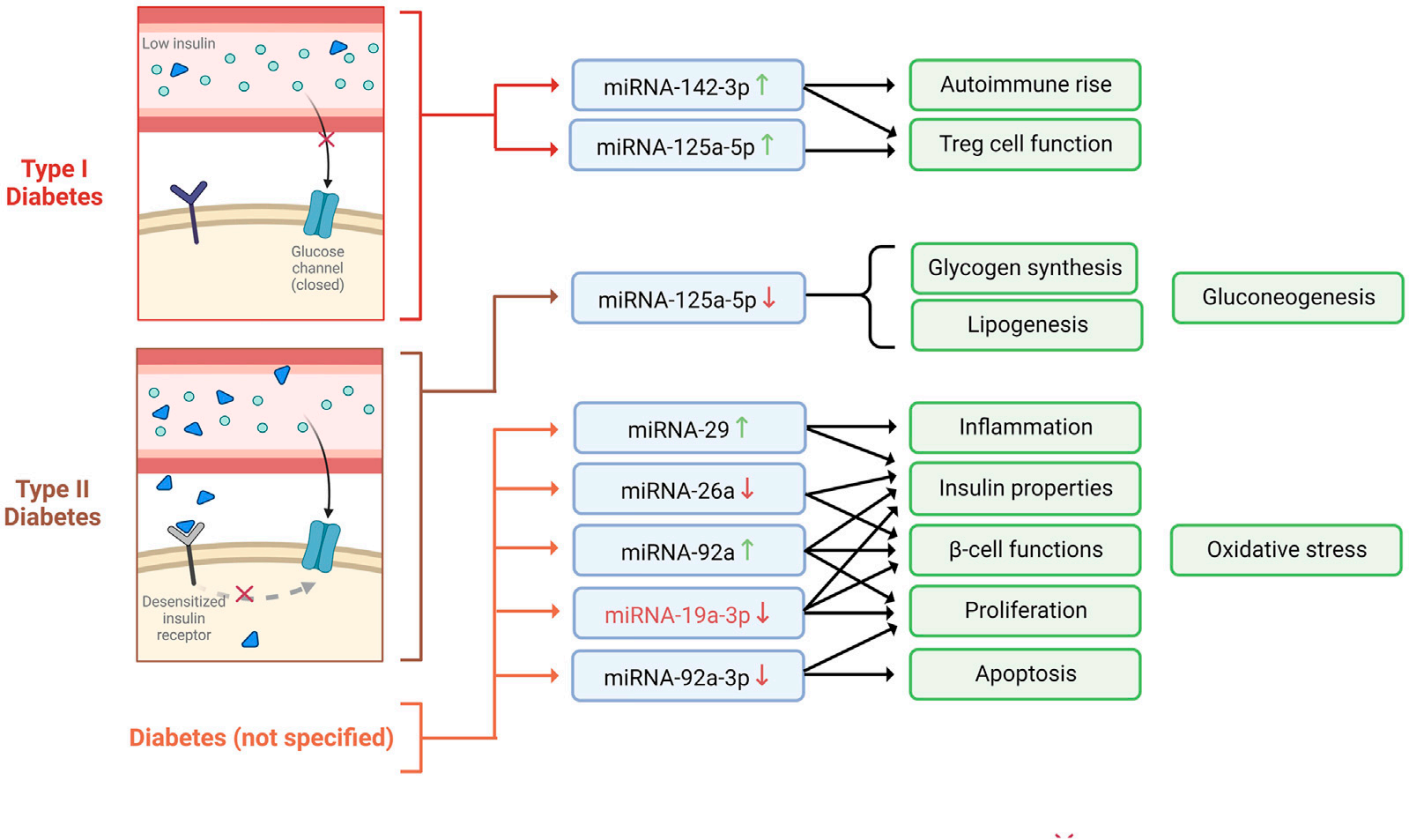
miRNAs expression profiles observed in diabetes mellitus type I, type II, and unspecified, and the biological mechanisms involved.

Diabetes mellitus (DM) is a chronic metabolic disorder characterized by persistently elevated blood glucose levels or hyperglycemia (Wang et al., 2020). Hyperglycemia can be caused by inefficiency in insulin action (also known as insulin resistance), secretion (also known as insulin deficiency), or both, as well as perturbations in the metabolism of carbohydrates, fats, or proteins (Cannata et al., 2020). The progression and development of DM are known to be a consequence of malfunctioned pancreatic *β*-cells, given that these cells are involved in insulin production (Wang et al., 2020). Type 1 (T1D) and type 2 diabetes (T2D) are the most common types of diabetes (Wang et al., 2020). T1D is caused by the autoimmune destruction of *β*-cells, leading to complete insulin deficiency. In contrast, T2D is attributed to a continuous loss of *β*-cell insulin secretion, often in the context of insulin resistance (Association, 2019). Diabetes prevalence increases yearly, affecting around 500 million people worldwide (Saeedi et al., 2019). According to the World Health Organization (2021), 1.5 million deaths were estimated to be caused directly by diabetes, placing this disease as the ninth leading cause of death worldwide. Given the high occurrence of DM and its complication, several investigations have been carried out to establish the role of miRNAs during the development of this disease to propose novel therapeutic approaches (Table 1) (Figure 1).

**TABLE 1:** Dysregulated miRNAs in endocrine disorders.

Endocrine disorder	Associated miRNA	miRNA target	Biological mechanism	Source	References
Diabetes mellitus	miRNA-92a↑	KLF2	Oxidative stress, high-glucose-induced *β*-cell apoptosis, insulin secretion, proliferation, *β*-cell protection	Pancreatic and lung mice tissue	Wang et al. (2018)
	miRNA-92a-3p↓		Proliferation in acute pancreatitis and apoptosis	Pancreatic rat tissue	Ling et al. (2020)
	miR-19a-3p↓	SOCS3	Proliferation, insulin secretion, *β*-cell apoptosis	Blood from diabetic patients	Li et al. (2016)
	miRNA-125a-5p↑	TNFR2	Treg cell function	Peripheral blood and lymph nodes of T1D patients	Sebastiani et al. (2017)
		CCR2			
	miR142-3p↑	TET2	Treg cell induction and stability, autoimmune activation, and progression	Human and mice CD4^+^ cells during the onset of islet autoimmunity and T1D	Scherm et al. (2019)
	miR-125a-5p↓	No reports	Glycogen synthesis, hepatic gluconeogenesis and lipogenesis	T2D mice and rat liver tissue	Xu et al. (2018)
		STAT3		Hepatocytes	
	miR-29↑	TRAF3	Insulinitis, insulin resistance and secretion, pancreatic inflammation	β cells of βTG mice	Sun et al. (2021)
	miR-26a↓	*CACNA1C, CTGF, CREBRF, ESR1, EXT1, MTPN, ONECUT2, PJA2, PLCB1, PFKFB2, RHOQ, SOX5, INSR, PDX1, AKT1, IGF1R*	Insulin sensitivity, preservation of *β*-cell function	Serum exosomes of obese mice and humans	Xu et al. (2020)
Thyroid diseases	miR-Let7d-5p↑	No reports	Immune response	Thyroid tissue from AITD patients	Martínez-Hernández et al. (2018)
	miR-21-5p ↑				
	miR-96-5p ↑				
	miR-142-3p ↑				
	miR-301a-3p				
	miR-223-3p				
	miR-301a-3p				
	miR-338-5p				
	miR-766-3p				
	miR-21-5p↑	No reports	Ciliogenesis	Thyroid tissue from AITD patients	Martínez-Hernández et al. (2019)
	miR-146b-3p↑				
	miR-5571-3p↑				
	miR-6503-3p↑				
	Let-7e	*IL-10*	Immune response	Peripheral blood mononuclear cells from HT and GD patients	Kagawa et al. (2016)
	miR-16-1-3p↑	No reports	Hormone regulation, tumor proliferation migration and agiogenesis	Plasmatic cells from GD patients	Yao et al. (2019)
	miR-122-5p↑				
	miR-221-3p↑				
	miR-762↑				
	miR-144-3p↓				
Osteoporosis	miR-133a↑	RANKL	Osteoclastogensis	Serum samples and lumbar spine BMD of post-menopausal osteoporotic patients	Li et al. (2018)
	miR-483-5p↑	*IGF2*	Osteoclast differentiation and apoptosis	Blood and bone tissue from patients with osteoporosis	Li et al. (2020)
	miR-133b↓	*GNB4*	Cell viability, apoptosis and osteoblast differentiation	Human osteoblasts	Wang et al. (2021)
	miR-148a-3p↑	*KDM6b*	Formation of adipocytes, osteogenic and macrophage differentiation, bone development	Mice bone marrow MSCs and stromal ST2 cell lines during adipogenesis	Tian et al. (2017)
	miR-19a-3p↓	*HDAC4*	Osteogenic differentiation	Osteoporosis patients	Chen et al. (2019)
	miR-449b-5p↓	*SATB2*	Osteoblast differentiation	Primary rat MSC cells	Li et al. (2019)
	miR-375↑	RUNX2	Osteogenesis, osteoblastogenesis. Osteoporosis dignosis	Serum from post-menopausal osteoporotic patients	Du et al. (2015); Kerschan-Schindl et al. (2021); Lei et al. (2019); Sun et al. (2017)
	miR-203a-3p↑	LRP5, β-catenin			
	miR-127-3p	BMP-2			
	miR-133b	DLX5			
	miR-214-3p	Osteorix			
	miR-31-5p				
	miR-143-3p				
	miR-188-5p				
	miR-582-5p				
	miR-550a-3p				
	miR-152-3p				
	miR-141-3p				
	miR-144-5p				
	miR-17-5p				
	miR-19b-3p				
	miR-29-3p				
	miR-320a				
	miR-335-5p				
	let-7b-5p				
	miR-1271-5p↑	FOXO1	Obsteoblast proliferation, osteoclast, and osteoblast differentiation, oxidative stress	Mice PBMC	Lu et al. (2021)
	miR-132-3p↑				
	miR-153a-3p↑				
	miR-153b-3 pA↑				
	miR-153-3p↑				
	miR-15a-5p↑				
	miR-15b-5p↑				
	miR-182-5p↑				
	miR-223-3p↑				
	miR-27a-3p↑				
	miR-370-3p↑				
	miR-486-5p↑				
	miR-9-5p↑				
Pituitary tumors	miR-448↓	*BCL2*	Metastasis	Human pituitary tumor tissue	He et al. (2020)
			Proliferation, migration, apoptosis	HP75 and MMQ tumor cell lines	
	miR-1↓	*G6PD*	Metastasis, glycolysis	Human pituitary tumor tissue	He et al. (2018)
			Proliferation, apoptosis	HP75 and MMQ tumor cell lines	
	miR-146b-5p↓	EPHA7	APA metastasis, tumor size, temozolomide resistance	Human APA tissues	Lou et al. (2021)
			Metastasis, apoptosis, proliferation, migration, invasion, temozolomide resistance, cancer cell differentiation, survival and movement	GH3 tumor cell line	
	miRNA-93-5p↑	ATG7	Cabergoline resistance	DA-resistant human prolactinoma tissues	Wu et al. (2019)
			Apoptosis, autophagy	MMQ and GH3 tumor cell lines	
	miR-186↓	*SKP2*	DNA synthesis, proliferation, cell cycle progression	GH-producing human pituitary tumor cells and GH3 cell line	He et al. (2019)
	miR-134	VEGFA	Tumor cell’s invasiveness	Patients with non-functional pituitary neuroendocrine tumors	Wang et al. (2020)
		SDF-1α	Cell cycle transition, viability, proliferation	aT3-1 cell line	
	miR-143-3p↓	No reports	Tumor progression	Plasma from pituitary adenomas	Németh et al. (2019)
	miR-26b↓		Proliferation, metastasis	Peripheral blood samples from untreated NFPA patients	Lin et al. (2018); Zhang et al. (2021)
	miR-138↓		Proliferation, metastasis, prognosis		You et al. (2019); Zhang et al. (2021)
	miR-206↓		Proliferation, migration		Park et al. (2018); Zhang et al. (2021)
	miR-let-7e↓		Tumor growth		Han et al. (2018); Zhang et al. (2021)
Cushing’s disease	miR-182-5p↓	No reports	Autoimmune response modulation	Serum from CS patients	Vetrivel et al. (2021)
	miR-96-5p↓				
	miR-183-5p↓				
	miR-1247-5p↑	CYB5A	Androstenedione production, proliferation	Adrenal tissue from CS patients	Vetrivel et al. (2022)
	miR-379-5p↑				
Adrenal insufficiency	miR-7977↑	ARRB1, CD80, CEBPG, CMKLR1, CRP, ENTPD1, HIPK2, HRH4, IL16, JAG1, MAVS, P2RY2, SEMA3G, SEMA4C, SIRPA, SLC11A, THRB, VGLL3	Immune response	Peripheral blood samples from Adisson’s disease patients	Fichna et al. (2021)
	miR-455↓	SCARB1	Steroidogenesis	Adrenal gland tissue from patients with adrenal insufficiency	Bitetto et al. (2022)
	miR-125a↓				
Multiple endocrine neoplasia	miR-486-3p↓	FASN	Adrenocortical proliferation, adrenal tumorogenesis	Men1^+/−^ mice adrenal glands	Li et al. (2021)
	miR-3156-5p↓	MORF4L2	Growth induction, growth arrest	Serum from MEN1 patients	Kooblall et al. (2022)

^a^ Note: ↑ indicates upregulation; ↓ indicates downregulation.

For example, it has been shown that myeloid KLF2 overexpression exerts a protective effect in mice against insulin resistance; therefore, it is considered a crucial regulator of obesity and its sequelae, such as diabetes (Sweet et al., 2020). Interestingly, Wang et al., 2018 suggested that miRNA-92a can negatively regulate the expression of KLF2, which is thought to function *via* modulating the Notch signaling pathway. The authors also revealed that miRNA-92a was predominantly expressed in the pancreas and lungs in mice models, which was an indicator of the involvement of miRNA-92a in pancreatic function. Moreover, they validated that miRNA-92a might modulate oxidative stress, inhibit high-glucose-induced apoptosis of *β*-cells, increase insulin secretion and proliferation, and protect pancreatic *β*-cell function (Wang et al., 2018). Consistent with these findings, another analogous study highlighted that the downregulation of miRNA-92a-3p enhances KLF2 expression and inhibits apoptosis, thus promoting proliferation in acute pancreatitis in pancreatic rat tissue (Ling et al., 2020).

Over the past years, SOCS3 has been associated with glucose metabolism and diabetes progression, given that it is a crucial negative regulator of insulin signaling (Iqbal et al., 2020). Notably, Li et al. (2016) found an inverse correlation between SOCS3 plasma levels and miR-19a-3p, which led them to suggest the role of the miR-19a-3p/SOCS3 axis dysregulation in T2D development. Furthermore, they noticed that the expression of miR-19-3p was notably downregulated in diabetic patients’ blood and that there was an inverse correlation between blood glucose concentration and plasma miR-19a-3p level (Li et al., 2016). Further, the authors demonstrated that miR-19a-3p promotes cell proliferation and insulin secretion while inhibiting pancreatic *β*-cell apoptosis by directly targeting SOCS3 (Li et al., 2016).

It is well known that Regulatory T cells (Treg) are important regulators of peripheral immune tolerance and that the development of autoimmunity disorders, including T1D, can be due to their insufficiency (Visperas and Vignali, 2016). In this context, an upregulation of miR-125a-5p in Treg cells obtained from T1D patients’ peripheral blood and lymph nodes was noted, suggesting that this overexpression could impede Treg-cell function (Sebastiani et al., 2017). Moreover, miR-125a-5p targets TNFR2 and CCR2. Remarkably, CCR2 has been implicated in T1D pathogenesis, and its expression is thought to modulate Treg cell function. This data suggests that the upregulation of miR-125a-5p in Treg cells of T1D patients leads to a decrease in CCR2 expression, which might hinder the migration of critical immune cells to the pancreas required for the maintenance of peripheral tolerance (Sebastiani et al., 2017). Similarly, Scherm et al., 2019 demonstrated that by inhibiting miR-142-3p, Treg induction and stability could be enhanced. More precisely, the authors showed that miR-142-3p was upregulated in both human and murine models with islet autoimmunity and T1D. Furthermore, miR-142-3p was identified to affect FOXP3 CNS2-induced DNA methylation and directly target TET2, a modulator of DNA methylation, and is suggested to be crucial to T cell activation and Treg induction. Overall, it has been concluded that the miR142-3p/TET2/FOXP3 axis hampers efficient Treg cell induction, compromising Treg stability and contributing to autoimmune activation and progression (Scherm et al., 2019).

Dysregulated glycolipid metabolism is one of the crucial causes of T2D development (Zhang et al., 2020). In this sense, Xu et al. (2018) showed that the enhancement of miR-125a-5p expression appears advantageous for glycolipid metabolism in T2D mice. Putatively, the authors evidenced a downregulation of miR-125a-5p in mouse and rat livers with T2D. Also, they demonstrated that miR-125a-5p targets the STAT3 in hepatocytes. Due to the involvement of STAT3 in glycolipid metabolism, it was revealed that the inhibition of miR-125a-5p increased hepatic gluconeogenesis and lipogenesis, decreased glycogen synthesis, and aggravated hyperglycemia and hyperlipidemia. Conversely, the elevation of miR-125a-5p mitigated glucose and lipid metabolic disorders. This information suggests a critical association between miR-125a-5p and glycolipid metabolism (Xu et al., 2018).

Pancreatic inflammatory diseases, including acute or chronic pancreatitis, could result in diabetes (Gál et al., 2021). Pancreatic inflammation has been found to be promoted by the overexpression of miR-29 in *β* cells of βTG mice (Sun et al., 2021). Explicitly, miR-29 overexpression causes the suppression of the TRAF3, whose axis regulates the response to metabolic stress associated with inflammation. Additionally, the inhibition of miR-29 improves insulitis, insulin resistance, and dysfunction of glucose-stimulates insulin secretion, causing the attenuation of inflammation and diabetes (Sun et al., 2021).

Given the importance of insulin secretion and sensitivity in maintaining normal glucose levels, their disruption results in DM (Bełtowski et al., 2018). In this regard, miR-26a has been shown to improve peripheral insulin sensitivity and preservation of *β* cell function (Xu et al., 2020). Putatively, miR-26a expression was downregulated in the serum exosomes of obese mice and humans. Interestingly, by triggering miR-26a expression, glucose dysregulation (hyperinsulinemia) could be averted since target genes of miR-26a such as *CACNA1C, CTGF, CREBRF, ESR1, EXT1, MTPN, ONECUT2, PJA2, PLCB1, PFKFB2, RHOQ*, and *SOX5*, are the regulators involved in insulin secretion and *β* cell proliferation/survival (Xu et al., 2020). Additionally, other target genes such as *INSR, PDX1, AKT1,* and *IGF1R* (β cell hyperplasia activators) were downregulated in the islet of RIP TG mice (Xu et al., 2020).

Altogether, the above information proves that the dysregulation of a great number of miRNAs contributes to the development and progression of diabetes. Therefore, these miRNAs could be potential biomarkers and therapeutic tools for treating diabetic patients.

### 1.2 Thyroid diseases

**FIGURE 2:**
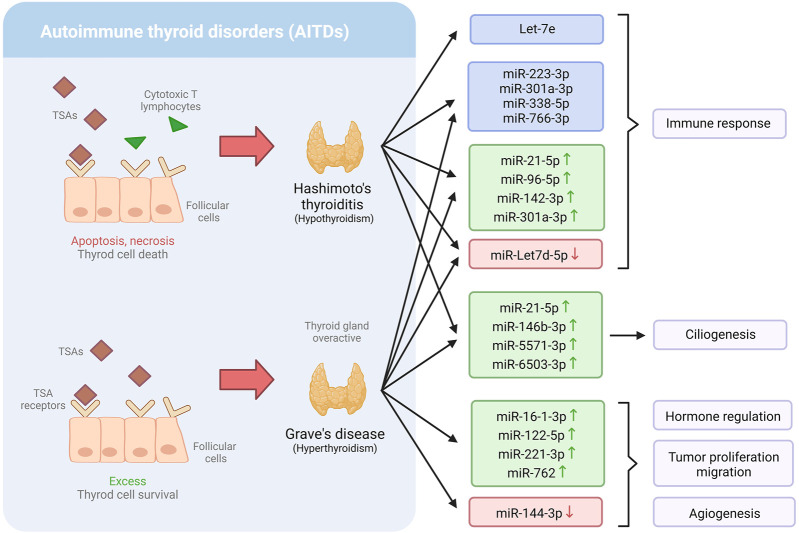
Dysregulation of miRNA expression profiles in Hashimoto’s thyroiditis and Grave’s disease, and the biological mechanism involved.

Thyroid diseases are conditions in which the thyroid gland is affected. This gland plays an essential role in regulating various metabolic processes (Kyritsi and Kanaka-Gantenbein, 2020). Thyroid diseases include hypothyroidism, hyperthyroidism, and autoimmune thyroid disorders (AITDs), such as Graves’ disease (GD) and Hashimoto’s thyroiditis (HT). According to the American Thyroid Association (2016), about 20 million Americans suffer from thyroid problems, and up to 60% of them are unaware of their condition. MiRNAs have been discovered to regulate numerous biological processes, including immune activation, making them promising candidates for the management of thyroid problems (Table 1) (Figure 2) (Martínez-Hernández et al., 2019).

Martínez-Hernández et al. (2018) found that individuals with AITDs have severely dysregulated levels of a variety of miRNAs connected to critical immunological mechanisms that might be involved in thyroid disorders. Eight miRNAs were found to be differentially expressed (miR-21-5p, miR-142-3p, miR-146a-5p, miR-146b-5p, miR-155-5p, miR-338-5p, miR-342-5p, and miR-766-3p) in patients with AITD. Furthermore, when the expression of these miRNAs was compared to additional miRNAs previously linked to AITD in peripheral blood, the expression of four of them (miR-Let7d-5p, miR-21-5p, miR-96-5p, miR-142-3p, and miR-301a-3p) was found to be notably higher in AITDs and, in patients with GD. Only miR-Let-7d was downregulated. Besides, the expression level of those miRNAs was associated with higher disease severity, including active ophthalmopathy, higher antibody titers, goiter, and/or higher recurrence rates. Interestingly, prior research has linked miR-Let7d-5p, miR-126-3p, miR-142-5p, miR-223-3p, and miR-301a-3p to autoimmune illnesses, including in AITD. Furthermore, the authors found a link between miR-Let7d-5p, miR-21-5p, miR-96-5p, miR-142-3p, and miR-301-3p and the levels of all three thyroid autoantibodies, suggesting that these miRNAs could be used to predict the likelihood of getting AITD (Martínez-Hernández et al., 2018).

Primary cilia are a sensory organelle that reacts to mechanical and chemical stimuli in the environment and transmits that information to the inside of the cell. Martínez-Hernández et al. (2018) examined the distribution and length of primary cilia, which act as sensory regulators in the thyroid to modulate hormone secretion in thyroid tissues from AITD patients, finding that both the number and length were significantly lower in AITDs than in healthy tissue. It was noticed that certain miRNAs, including miR-21-5p, miR-146b-3p, miR-5571-3p, and miR-6503-3p, were upregulated in the cilia, therefore, associated with these conditions. Therefore, the study suggests the potential use of miRNAs to detect AITD (Martínez-Hernández et al., 2019).

*IL-10* is a cytokine directly involved in reducing inflammation induced by macrophages and T cells. One of the essential functions of *IL-10* is inhibiting Th1 cells, a type of T cell that synthesizes inflammatory cells (Ouyang and O’Garra, 2019). Interestingly, Let-7e has been found to target the interleukin *IL-10* genes (Kagawa et al., 2016). Precisely, Kagawa et al. (2016) observed that *IL-10* production was inhibited in peripheral blood mononuclear cells of patients with HT and that serum levels of Th1 cytokines were elevated. Importantly, let-7e expression was considerably higher in HT patients compared to GD patients or control subjects. However, there were no significant variations in Let-7e expression levels between GD patients and control subjects. Moreover, Let-7e expression levels in severe HT patients were found to be considerably lower than in mild HT patients (Kagawa et al., 2016). These findings lead to the understanding of Let-7e in the regulation of the immune response of people with HT, which might eventually help with HT diagnosis and treatment.

Graves’ disease is an autoimmune condition known as the most common cause of hypothyroidism (Yao et al., 2019). It is characterized by an activated immune system, which results in the production of thyroid-stimulating antibodies (TSAs); consequently, it makes the thyroid gland overactive, producing more hormones than usual (Razmara et al., 2021). GD is a complex condition resulting from environmental and genetic factors (including mutations in the *TSHR* and *CD40* genes). Yao et al. (2019) demonstrated that various plasmatic miRNAs are associated with GD and might serve as biomarkers for diagnosing this condition. They found that five miRNAs were substantially differentially expressed in GD. Among them, miR-16-1-3p, miR-122-5p, miR-221-3p, and miR-762 were upregulated, while miR-144-3p was downregulated. Intriguingly, miR-762 was positively associated with FT3, a thyroid hormone, and TRab (Yao et al., 2019), which is considered the gold standard diagnostic test for the autoimmunity of GD (Bell et al., 2018). Moreover, miR-144-3p has been shown to be downregulated in many kinds of carcinoma and is associated with tumor proliferation, migration, and angiogenesis (He et al., 2020).

Since thyroid diseases affect a large number of people, most of whom are unaware of it, it is crucial to find new ways to detect thyroid diseases to implement an adequate early treatment. MiRNAs have proven to be a valuable tool for the early detection of thyroid diseases such as HT and GD; however, further research is required to use them as potential disease biomarkers. Moreover, investigating the roles of miRNAs in hyper and hypothyroidism might open a new arena of thyroid disease management.

### 1.3 Osteoporosis

**FIGURE 3:**
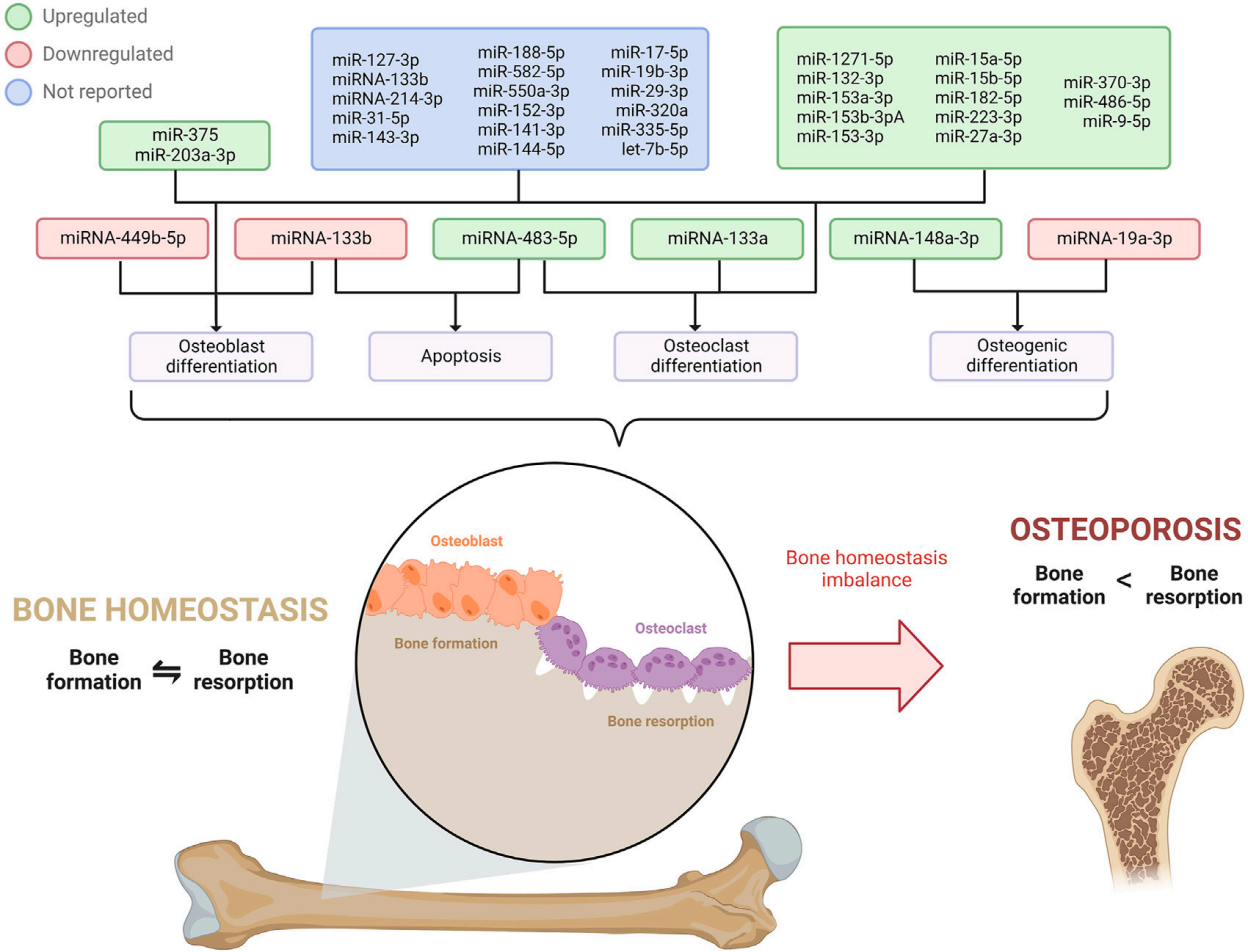
miRNAs expressions profile involved in osteoblast, osteoclast, and osteogenic differentiation, and apoptosis that disrupts bone homeostasis, ultimately leading to osteoporosis.

Osteoporosis is a multifactorial condition considered as the most prevailing metabolic bone disorder in humans (Scimeca et al., 2017). It is caused by complex interactions between genetic and environmental factors (Li et al., 2018) and is characterized by an imbalance between the bone formation mediated by osteoblasts and the bone resorption mediated by osteoclasts. Bone homeostasis is commonly altered by dysregulation in osteoblasts and osteoclasts’ proliferation, differentiation, or apoptosis (Wang et al., 2021), which leads to bone mass and strength decrease, degenerative change of bone microarchitecture, and non- or low-trauma fractures (Cosman et al., 2014; Scimeca et al., 2017). Osteoporosis usually has no symptoms prior to a first fracture, which is associated with a risk increment of a subsequent fracture by 86% (International Osteoporosis Foundation, 2022). Osteoporosis has become a serious health concern, affecting around 50% of post-menopausal females and 20% of males over 50 (Gennari et al., 2016). Furthermore, it causes over 8.9 million fractures annually (Hu et al., 2018), many of which lead to mortality (International Osteoporosis Foundation, 2022). MiRNAs have an important role in the regulation of bone formation and resorption (Li et al., 2018; Chen et al., 2019) that allows a balance to maintain the healthy state of the bones, and their dysregulation is associated with osteoporosis; however, the precise underlying mechanism is still poorly understood. Hence, there is a current need to elucidate the regulatory pathways of miRNAs in osteoporosis to prevent and treat this global disease (Table 1) (Figure 3).

It is estimated that one in three 50 + aged women will suffer a fracture in their remaining lifetime (International Osteoporosis Foundation, 2022), strongly influenced by estrogen deficiency and aging (Li et al., 2018). One of the hallmarks of post-menopausal osteoporosis is a low bone mineral density (BMD) derived from the altered activity of osteoblasts and osteoclasts. Notably, miR-133a has been significantly upregulated in serum samples and showed a negative correlation with lumbar spine BMD of post-menopausal patients with osteoporosis. Interestingly, this miRNA was upregulated during osteoclastogenesis, thus promoting the differentiation of RAW264.7 and THP-1 cells into osteoclasts, which would be induced by RANKL, a crucial cytokinin for osteoclast development (Li et al., 2018). Consistently, an *in vivo* analysis in OVX rats demonstrated that a knockdown of miR-133a augmented lumbar spine BMD, dysregulated the serum levels of factors related to osteoclastogenesis, and modified the histomorphology of the bone mainly by perforation, connectivity loss, and thinning of the trabecular bone. Altogether, it was demonstrated that miR-133a promotes osteoclast differentiation, thus contributing to the regulation of osteoporosis in post-menopausal women (Li et al., 2018).

Similarly, miR-483-5p has also been suggested to promote osteoclast differentiation in osteoporosis. Putatively, in samples of blood and bone tissue from osteoporotic patients, miR-483-5p was found to be significantly upregulated, whereas its predicted target *IGF2* was underexpressed. Interestingly, the upregulation of miR-483-5p promoted the differentiation of the M-CSF, a relevant cytokine for osteoclast differentiation in mammals, and RANKL-induced CD14^+^ peripheral blood mononuclear cells into osteoclasts. Moreover, overexpression of *IGF2* was discovered to revert the miRNA effects on osteoclast differentiation and thus enhance osteoclast apoptosis. Therefore, a dysregulation in miR-483-5p expression might contribute to osteoclast differentiation by targeting *IGF2*, causing dysregulation in the balance between bone formation and resorption, ultimately causing osteoporosis (Li et al., 2020).

The bone formation also relies on osteoblast differentiation (Wang et al., 2021); therefore, an alteration in this process may lead to skeletal disorders such as osteoporosis. A study in human osteoblasts revealed the downregulation of miR-133b and subsequent overexpression of its target *GNB4* in osteoporotic patients (Wang et al., 2021). Intriguingly, the induction of miR-133b led to higher cell viability, diminished apoptosis, and promotion of osteoblast differentiation (Wang et al., 2021); hence, the underlying mechanism of the miR-133b/*GNB4* might be of use for osteoporosis treatment. Correspondingly, miRNAs can also influence bone marrow mesenchymal stem cells (MSCs), known to be osteoblast and adipocyte progenitor cells (Hu et al., 2018). Since an imbalance of osteoblast and adipogenic differentiation of MSCs generates a bone mass decrease associated with osteoporosis (Hu et al., 2018; Li et al., 2019), the regulation mechanism of miRNAs in these processes has been extensively studied in order to develop potential target treatments for this skeletal disorder. In that context, miR-148a-3p was overexpressed in mice bone marrow MSCs and stromal ST2 cell lines during adipogenesis (Tian et al., 2017). miR-148a-3p potentiated the formation of adipocytes in the adipogenic medium while inhibiting osteoblast differentiation. Further study revealed *KDM6b* as the direct target of miR-148a-3p. In the investigation, *KDM6b* was found to influence the differentiation into mature adipocytes; however, it has also been related to the modulation of osteogenic commitment of MSCs, osteogenic and macrophage differentiation, and bone development. Altogether it was concluded that miR-148a-3p has a positive adipogenic and negative osteogenic function by directly targeting and negatively regulating *KDM6B*, affecting the MSCs fate decision (Tian et al., 2017).

Although bone mass recovery strategies are limited, miRNA-mediated osteogenic differentiation has become a target of interest for osteoporosis therapeutics. In that matter, Chen et al. (2019) discovered that serum levels of miR-19a-3p were underexpressed in patients with osteoporosis. Furthermore, the levels of miR-19a-3p, *RUNX2,* and *OCN* (these last being osteogenesis-related genes) would be gradually upregulated as the osteogenic differentiation of MSCs prolongated. miR-19a-3p was found to target the *HDAC4* gene, a critical factor in bone formation, bone metabolism, and osteoblast differentiation. Thus, the analyzed miRNA was suggested to reduce osteoporosis progression by accelerating the osteogenic differentiation of MSCs by inhibiting *HDAC4* (Chen et al., 2019). Li et al. (2019a) consistently studied miR-449b-5p expression in primary rat MSC cells, and miR-449b-5p was reported to be underexpressed in osteogenic differentiation; moreover, the differentiation could be inhibited by the miRNA overexpression, thus revealing the mechanism of miR-449b-5p in suppressing bone formation. In addition, miR-449b-5p was proved to target the *SATB2* gene, which enhances osteoblast differentiation, regulates skeletal development, and promotes bone formation. Therefore, miR-449b-5p is thought to repress the osteogenic differentiation of MSCs *via SATB2*, therefore promoting the progression of osteoporosis (Li et al., 2019).

Kerschan-Schindl et al. (2021) used a signature panel of nineteen circulating miRNAs to serve as biomarkers to diagnose osteoporosis in post-menopausal patients based on the WHO criteria and with the ability to identify patients that had previously suffered fractures. The miRNAs in the panel represent different independent factors related to bone disease and the risk of fractures. The panel includes miR-127-3p, miR-133b, miR-203a, miR-214-3p, miR-31-5p, and miR-375 (all of which the effects of hemolysis did not bias measurement); miR-143-3p, miR-188-5p, and miR-582-5p (these miRNAs along with miR-375 strongly contributed with the WHO criteria); miR-550a-3p and miR-152-3p (which contributed to the major osteoporotic fractures); and miR-141-3p, miR-144-5p, miR-17-5p, miR-19b-3p, miR-29-3p, miR-320a, miR-335-5p, and let-7b-5p. Out of these, the upregulation of serum miR-375 indicated the presence of osteoporosis according to the WHO criteria and has been associated with reducing the bone-formation effect of teriparatide, a drug to treat severe osteoporosis (Lei et al., 2019). MiR-375 has been found to negatively modulate osteogenesis (Kerschan-Schindl et al., 2021), possibly by targeting RUNX2 (Du et al., 2015; Lei et al., 2019), and suppressing *WNT* pathways by targeting LRP5 and ß-catenin (Sun et al., 2017). Moreover, the upregulation of miR-203a-3p was a potential marker for fragility fractures and has been identified to negatively regulate osteoblastogenesis *via* BMP-2 and DLX5, which activate the transcription factors for osteoblast differentiation: RUNX2 and Osterix. Altogether, the analyzed panel could serve as a novel and non-invasive tool for osteoporosis diagnosis and identifying patients with fracture history (Kerschan-Schindl et al., 2021).

Currently, microRNA biomarkers for osteoporosis are found to be very promising since conventional protein osteoporosis biomarkers are not very efficient and often produce false positive results. In this matter, Lu et al. (2021) found thirteen significantly consistent overexpressed miRNAs (miR-1271-5p, miR-132-3p, miR-153a-3p, miR-153b-3pA, miR-153-3p, miR-15a-5p, miR-15b-5p, miR-182-5p, miR-223-3p, miR-27a-3p, miR-370-3p, miR-486-5p, and miR-9-5p) in mice PBMC that were positively correlated with osteoporosis progression, therefore elucidating their potential role as biomarkers for this disease. The mentioned miRNAs were FOXO1 regulators, an important molecule in bone metabolism that activates osteoblasts’ proliferation and differentiation, suppresses the differentiation and viability of osteoclasts, and diminishes the negative effects of oxidative stress to favor bone cell function (Lu et al., 2021).

Although osteoporosis is a common concern in the elderly and post-menopausal population, bone restoration strategies are currently limited. Recently, several investigations have elucidated the role of miRNAs in osteoporosis which could be promising in discovering novel miRNA-based diagnostic and therapeutic strategies that could increase the patient’s quality of life.

### 1.4 Pituitary tumors

**FIGURE 4:**
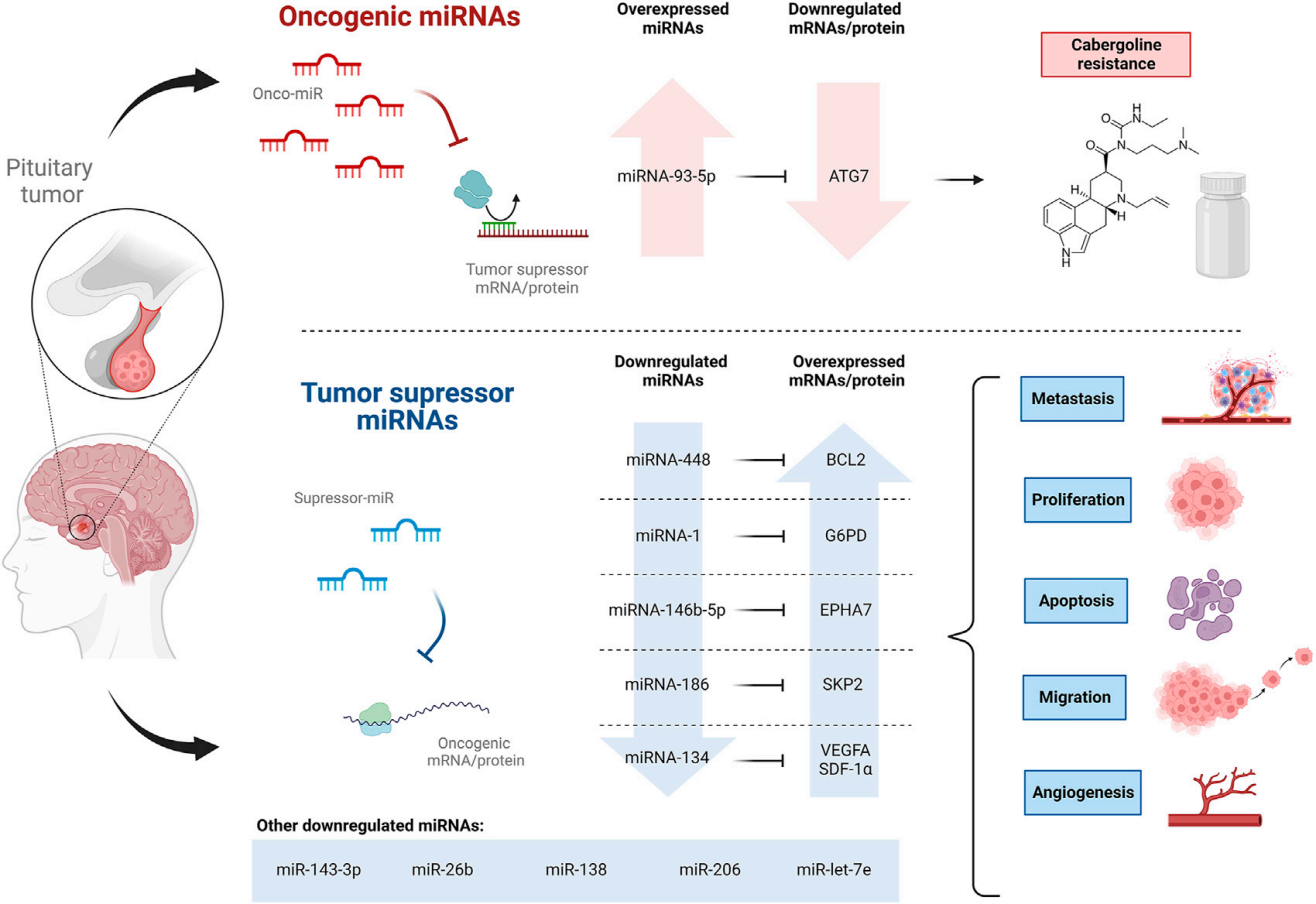
Oncogenic and tumor suppressor miRNAs expression profile involved in pituitary tumors, ultimately affecting cabergoline resistance, proliferation, metastasis, apoptosis, and migration.

Pituitary tumors, also called pituitary neuroendocrine tumors, are adenomas that arise from the anterior pituitary gland and constitute around 10 to 15 percent of intracranial tumors (Asa et al., 2017; Lou et al., 2021). Pituitary tumors arise sporadically from any of the five hormone-producing cell types of the adenohypophysis, but despite being monoclonal, they contain plenty of multi-responsive and multi-hormonal cells whose role is not yet fully understood (Fiordelisio et al., 2021). Many neoplasms, including pituitary tumors, have exhibited altered miRNA expression, thus being a significant field of research for understanding, diagnosing, and treating pituitary tumors (Table 1) (Figure 4).

Although pituitary tumors are mostly benign, they have the potential to develop into cancer. He et al. (2020) noticed a reduced expression of miR-448 in human pituitary tumor tissues and an even lower expression in tissues from patients with metastasis. The induced overexpression of miR-448 was evaluated in HP75 and MMQ cells (pituitary adenoma cell lines), showing inhibition of proliferation and migration of both cell lines and an increase in the apoptosis rate. It was suggested that miR-448 negatively regulates its target gene, *BCL2*, an integral outer mitochondrial membrane protein that inhibits apoptosis in HP75 and MMQ cells. In addition, increased levels of PARP, E-cadherin, caspase-3, and decreased levels of vimentin were also noticed in HP75 and MMQ cells overexpressing miR-448 (He et al., 2020). Caspase-3 and PARP are pro-apoptotic proteins (Hsu et al., 2018), while E-cadherin and vimentin are biomarkers that, in this case, suggest the inhibition of epithelial to mesenchymal transition of pituitary tumor cells (Liao et al., 2019; He et al., 2020). Hence, the elucidated miR-448–*BCL2* pathway might be of use as a therapeutic strategy to regulate the malignant behavior of pituitary tumor cells.

He et al. (2018) also observed the downregulation of miR-1 in human pituitary tumor tissues. Moreover, a much lower abundance of miR-1 was found in the presence of lymphatic metastasis, associated with a worse prognosis in patients. Other assessments showed that the overexpression of miR-1 in HP75 and MMQ cell lines suppresses the proliferation and increases the apoptosis rate of the cells. Also, it was demonstrated that miR-1 negatively regulates the protein expression of its target gene *G6PD*, limiting its function of generating ribose-5-phosphate and NADPH, which are promoters of glycolysis and nucleotide biosynthesis in tumor cells; therefore, pituitary tumor tissues under expressing miR-1 showed an augmentation of *G6PD* (C. He et al., 2018), suggesting miR-1 as a target to diagnose and treat pituitary tumors.

Pituitary tumors are referred to as aggressive pituitary adenomas (APAs) when they exhibit aggressive behaviors such as solid invasiveness and resistance to conventional therapies (Lou et al., 2021). The role of miRNA-146b-5p in APA was studied by Lou et al. (2021). MiRNA-146b-5p was found to be downregulated in human APA tissues and pituitary tumor cell lines (AtT-20, HP75, GH3, GT1-1) compared to non-tumor tissues and normal human astrocytes, respectively. Furthermore, the downregulation of miRNA-146b-5p in APA patients was associated with poor prognosis, poor survival rate, and features indicative of advanced tumor stage related to APA metastasis, such as temozolomide resistance, larger tumor size, poorer Hardy grade, and poorer Knosp grade (Lou et al., 2021); these last grades refer to classification systems indicating local invasion and cavernous sinus invasion (Araujo-Castro et al., 2021). Subsequent assessments demonstrated that the induced overexpression of miRNA-146b-5p in GH3 cells suppressed metastasis by triggering cell apoptosis and negatively regulating cell proliferation, migration, and invasion. The direct target of miRNA-146b-5p, EPHA7 was also found to be overexpressed in APA tissues as well as in the pituitary tumor cell lines previously mentioned (Lou et al., 2021). EPHA7 mediates the inflammatory response to cell injury, and its low expression has been found in different carcinomas (Gajdzis et al., 2020). The anti-metastatic properties of miRNA-146b-5p in GH3 cells were reversed by the induced overexpression of EPHA7 (Lou et al., 2021). Interestingly, compared to chemosensitive tissues, chemoresistant tissues showed overexpression of EPHA7 and underexpression of miRNA-146b-5p. The same results were found in a temozolomide-resistant cell line of GH3 compared to the parental cell line. It was speculated that miRNA-146b-5p might regulate autophagy since treatment with this miRNA decreased the autophagic activity of temozolomide-resistant cells, while Lv-EPHA7 treatment enhanced it. Thus, it was suggested that abnormal expressions of miRNA-146b-5p and EPHA7 might be involved in the temozolomide resistance in APA. Finally, IRAK4 and TRAF6 protein expression and NF-κB phosphorylation were suppressed in GH3 cells overexpressing miRNA-146b-5p; however, restoration of EPHA7 expression reversed these inhibitory effects. The IRAK4/TRAF6/NF-κB signaling pathway is essential in regulating cancer cell differentiation, survival, and movement, and EPHA7 is required. Hence, miRNA-146b-5p may inhibit invasion and metastasis by directly targeting EPHA7 and suppressing the IRAK4/TRAF6/NF-κB signaling pathway (Lou et al., 2021). These findings suggest that miRNA-146b-5p could be used as a molecular marker to evaluate and predict pituitary tumor aggressiveness and chemotherapeutic drug resistance.

Pituitary tumors can be functioning (which involves the overproduction of certain hormones) or non-functioning (low hormone production) (Butt and Srinivasan, 2022). The most common functioning pituitary tumors are prolactin-secreting tumors called prolactinomas (Kim et al., 2022). In most cases, cabergoline (CAB) or other dopamine agonists are used for the treatment, but there are dopamine agonist-resistant prolactinomas whose management remains a challenge (Wu et al., 2019). Wu et al. (2019) proposed that miRNA-93-5p, a miRNA overexpressed in dopamine agonists-resistant prolactinoma tissues, targets ATG7 and intervenes in CAB resistance. Autophagy is a crucial pathway for cell survival under stress that degrades large structures such as organelles and proteins (Li et al., 2019), and ATG7 is an essential protein downregulated in dopamine agonists-resistant human prolactinoma tissues (Wu et al., 2019). Assays performed with MMQ and GH3 rat prolactinoma cell lines indicated that the upregulation of miRNA-93-5p may decrease the CAB-induced autophagic cell death by inhibiting the expression level of cleaved caspase-8 and LC3-II, proteins related with CAB-induced apoptosis and autophagy, respectively. Afterward, the miRNA-93-5p inhibitor was used to transfect MMQ cells which were then injected subcutaneously into nude mice; the results showed an enhancement of the therapeutic effectiveness of CAB, a diminution in the prolactin expression in mice, an upregulation of LC3-II protein, and the overproduction of autophagosomes. Therefore, the investigation suggests that miRNA-93-5p inhibition enhances CAB efficiency *in vivo* by upregulating autophagy.

Growth hormone (GH) pituitary tumors are another subtype of functioning pituitary tumors characterized by excessive production of GH by the pituitary gland leading to acromegaly and gigantism (Ji et al., 2022). He et al. (2019) reported a reduced expression of miR-186 in GH-producing human pituitary tumors. The miR-186 regulates the expression of its direct target, the *SKP2*, an oncogene that targets *p27Kip1* to inhibit its expression (He et al., 2019). The *p27Kip1* is a tumor suppressor that inhibits the transition of the cell cycle from the G1 to the S phase (Li et al., 2021). The role of miR-186 was tested in human GH-secreting pituitary tumor cells and GH3 cells, and its inhibition resulted in the overexpression of *SKP2* and underexpression of *p27Kip1*, causing an induction in the DNA synthesis and cell proliferation. Consistently, the overexpression of miR-186 had the opposite effect on the cell lines and induced G0/G1 cell cycle arrest. In addition, miR-186 and p27Kip1 were downregulated in GH-producing human pituitary tumor tissues compared to normal human pituitary tissues, while *SKP2* was upregulated (He et al., 2019). The above-mentioned information indicates that miR-186 has a tumor-suppressive role in pituitary tumors since it modulates the cell cycle *via SKP2/p27Kip1*, thus revealing a novel mechanism that might have significant therapeutic implications.

Non-functional pituitary neuroendocrine tumors remain a challenge since the only first-line treatment choices are surgery and radiotherapy, and early detection of invasiveness is scarce due to a lack of diagnostic biomarkers (Bao et al., 2021). Wang et al. (2020) analyzed specimens of patients with non-functional pituitary neuroendocrine tumors and reported that miR-134 might inhibit the tumor cell’s invasiveness and expression of Ki-67, a marker of cellular proliferation. The verified target of miR-134 was the VEGFA (Wang et al., 2020), the most functional isoform of the proangiogenic factors VEGF, which contribute to angiogenesis, successively helping the generation and development of cancer (Zhang et al., 2019). Additionally, they found that by influencing the expression of miR-134, SDF-1α could promote the development of tumors. SDF-1α and VEGFA expression levels were relatively high in the analyzed invasive specimens, defining invasiveness as resistance to conventional treatment and numerous recurrences. It was confirmed in mouse pituitary aT3-1 cells that due to miR-134 upregulation, VEGFA expression and transition of the cell cycle from the G1 to the S phase are inhibited. Interestingly, SDF-1α treatment reduces miR-134 expression and boosts VEGFA expression, which promotes cell viability and proliferation by allowing cell cycle transition (Wang et al., 2020). For that reason, the SDF-1α/miR-134/VEGFA axis might be a potential target for detecting and treating non-functional pituitary neuroendocrine tumors.

With the objective of identifying circulating miRNAs that could serve as biomarkers for pituitary adenomas, Nemeth et al. (2019) investigated the dysregulation of several miRNAs in plasma and extracellular vesicles from patients with pituitary adenomas at a preoperative, as well as early and late postoperative stages. A total of 29 miRNAs were found promising to distinguish preoperative plasma samples from normal controls. Interestingly, the study demonstrated that miR-143-3p was substantially differentially expressed (downregulated) in late postoperative samples of FSH/LH + adenomas compared to preoperative samples, indicating a successful surgery. Nevertheless, no significant alteration of this miRNA has been noticed when comparing early postoperative samples with preoperative ones. Although the results are promising, further investigation is needed to determine the potential role of this miRNA as a marker for tumor recurrence (Németh et al., 2019).

Dysregulation of miRNAs has also been associated with tumor incidence and progression, although the regulatory mechanism of these tiny molecules behind non-functional pituitary adenomas (NFPA) has not yet been clarified. In this context, Zhang et al. (2021) explored the correlation between miRNAs expression levels and NFPA by comparing miRNA expression levels in peripheral blood samples from untreated NFPA patients and healthy controls. Four specific miRNAs: miR-26b, miR-138, miR-206, and miR-let-7e were significantly downregulated in NFPA patients than healthy controls, suggesting the involvement of these molecules in the occurrence and progression of NFPAs. Relevantly, miR-26b has been previously associated with the inhibition of proliferation and metastasis in hepatocellular carcinoma (Lin et al., 2018); miR-138 has been reported as a tumor-suppressor gene related to proliferation, metastasis, and prognosis of colon cancer cells (You et al., 2019); miR-206 has been reported to increase the migration and proliferation of colorectal cancer cells (Park et al., 2018); and miR-let-7e has been reported to diminish tumor growth (Han et al., 2018). Altogether this information could imply using these four miRNAs as novel targets for the clinical management of NFPA (Zhang et al., 2021).

Because of their essential roles in regulating the development of pituitary tumors, miRNAs have significant clinical value. More specifically, miRNAs could be used to diagnose the presence and progression of a tumor and classify it more accurately, predict invasiveness or aggressiveness, treat or regulate the behavior of pituitary tumors, and evaluate the success of an operation.

### 1.5 Other endocrine disorders

#### 1.5.1 Cushing’s disease

Cushing’s syndrome (CS) is a rare disease caused by an overproduction of cortisol in most cases due to adrenocorticotropic hormone (ACTH)-producing pituitary adenomas (Belaya et al., 2020). In a study where circulating miRNA expression profiles were compared in serum samples of patients with and without CS, miR-182-5p was identified as a potential biomarker due to its persistent considerable differential expression between the samples (Vetrivel et al., 2021). Along with miR-182-5p, other members of the miR-183 cluster, including miR-96-5p and miR-183-5p, were found to be substantially downregulated in the samples. This cluster is associated with auto-immune, neurological, and psychiatric disorders, as well as with cancer (Vetrivel et al., 2021). In a recent study, upregulation of miR-1247-5p and miR-379-5p was observed in the adrenal tissues of patients with different forms of CS in comparison to controls (Vetrivel et al., 2022). One target of miR-1247-5p and miR-379-5p was found to be Cytochrome b5 (CYB5A), which was notably downregulated in all forms of CS and is known to intervene in the regulation of androstenedione production. However, other target genes were identified as involved in the WNT signaling pathway, whose dysregulation promotes proliferation in ACTH-secreting pituitary adenomas (Vetrivel et al., 2022).

#### 1.5.2 Adrenal insufficiency

Primary adrenal insufficiency (PAI), commonly known as Addison’s disease, is the consequence of the malfunction of the adrenal cortex that causes chronic glucocorticoid or mineralocorticoid deficiency (Barthel et al., 2019). PAI affects 1 in every 5,000–7,000 humans worldwide (Saverino and Falorni, 2020). Recent studies have proven the involvement of miRNAs in this condition. A significant upregulation of miR-7977 was found in CD4^+^ T cells obtained from the peripheral blood of patients with autoimmune Addison’s disease compared to healthy controls (Fichna et al., 2021). Additionally, a positive correlation was found between the amount of co-occurring autoimmune disorders and the abundance of miR-7977, as well as with the expression of miR-7977 and circulating autoantibodies against thyroid peroxidase (aTPO). Although the particular target of miR-7977 is still unknown, 18 potential targets involved in autoimmunity were identified, and they are ARRB1, CD80, CEBPG, CMKLR1, CRP, ENTPD1, HIPK2, HRH4, IL16, JAG1, MAVS, P2RY2, SEMA3G, SEMA4C, SIRPA, SLC11A, THRB, and VGLL3. These genes are thought to be involved in immune response, primary cellular component regulation, and metabolic processes (Fichna et al., 2021). Similarly, miRNA-455 and miRNA-125a were significantly downregulated in the adrenal gland of Allgrove syndrome patients with adrenal insufficiency (Bitetto et al., 2022). These miRNAs are known to be involved in the regulation of Scavenger receptor class B-1 (SCARB1), an important factor in steroidogenesis found to be downregulated in the samples. Due to the downregulation of miR-455 and miR-125a, there is an induction of SCARB1 on the cell surface (Bitetto et al., 2022).

#### 1.5.3 Multiple endocrine neoplasia

Multiple endocrine neoplasia type 1 (MEN1) is an autosomal dominant disorder characterized by various endocrine and non-endocrine tumors. It is a rare disorder, given that it affects between 3–20 in every 100,000 people (Brandi et al., 2021). Recently, miR-486-3p was found to be notably downregulated in Men1^+/−^ mice’s adrenal glands (Li et al., 2021). miR-486-3p was proven to target fatty acid synthase (FASN), which is involved with adrenocortical proliferation. Thus, it is theorized that miR-486-3p downregulation might play a crucial role in adrenal tumorigenesis (Li et al., 2021). Moreover, a significant downregulation of miR-3156-5p was observed in serum samples of MEN1 patients compared to controls. This downregulation may be the result of a reduction in MEN1 expression (Kooblall et al., 2022). It was also found that miR-3156-5p directly targets MORF4L2, given that miR-3156-5p upregulation causes a decrease of MORF4L2 expression, which plays an important role in the activation of oncogene and proto-oncogene-mediated growth induction, as well as in tumor suppressor-mediated growth arrest. Additionally, MORF4L2 is part of the NETest, a useful tool for neuroendocrine tumor (NET) subtype management and diagnosis, therefore, it might be of utility for MEN1 diagnosis (Kooblall et al., 2022).

## 2 Discussion

Endocrine disorders are a significant health issue that decreases the quality of patients’ life and sometimes could be fatal. To date, none of the conventional diagnoses or therapies for endocrine disorders are efficient or precise, which has outstood the urge to develop novel and more functional techniques. Recently, miRNAs have been shown to regulate various vital processes associated with endocrine disorders such as diabetes mellitus, thyroid diseases, osteoporosis, pituitary tumors, Cushing’s syndrome, adrenal insufficiency, and multiple endocrine neoplasia. Although the precise roles of several miRNAs in various signaling pathways during the development of endocrine disorders have yet not been fully elucidated, these small molecules have drawn the attention of global researchers to be used for novel therapeutic purposes. Furthermore, miRNAs have shown promising results as disease biomarkers to detect the disease at an early stage, distinguish among different conditions of the patients, as well as to determine the disease’s severity. Altogether, miRNAs have revealed their potential for developing novel tools and techniques that allow the precise detection and management of endocrine disorders, resulting in a general improvement in the patient’s life quality.

## Abbreviations

miRNA, microRNA; DM, Diabetes mellitus; T1D, Type 1 diabetes; T2D, Type 2 diabetes; Treg Regulatory T cells AITDs, Autoimmune thyroid disorders; GD, Graves’ disease; HT, Hashimoto’s thyroiditis; BMD, Bone mineral density; MSC, Mesenchymal stem cells; APA, Aggressive pituitary adenoma; CAB, Cabergoline.
