# Supplementary figures and images for: Regulatory mechanisms of microRNAs in endocrine disorders and their therapeutic potential
Source: Front Genet. 2023 Feb 21;14:1137017. doi: 10.3389/fgene.2023.1137017 (PMC9989203; doi:10.3389/fgene.2023.1137017)

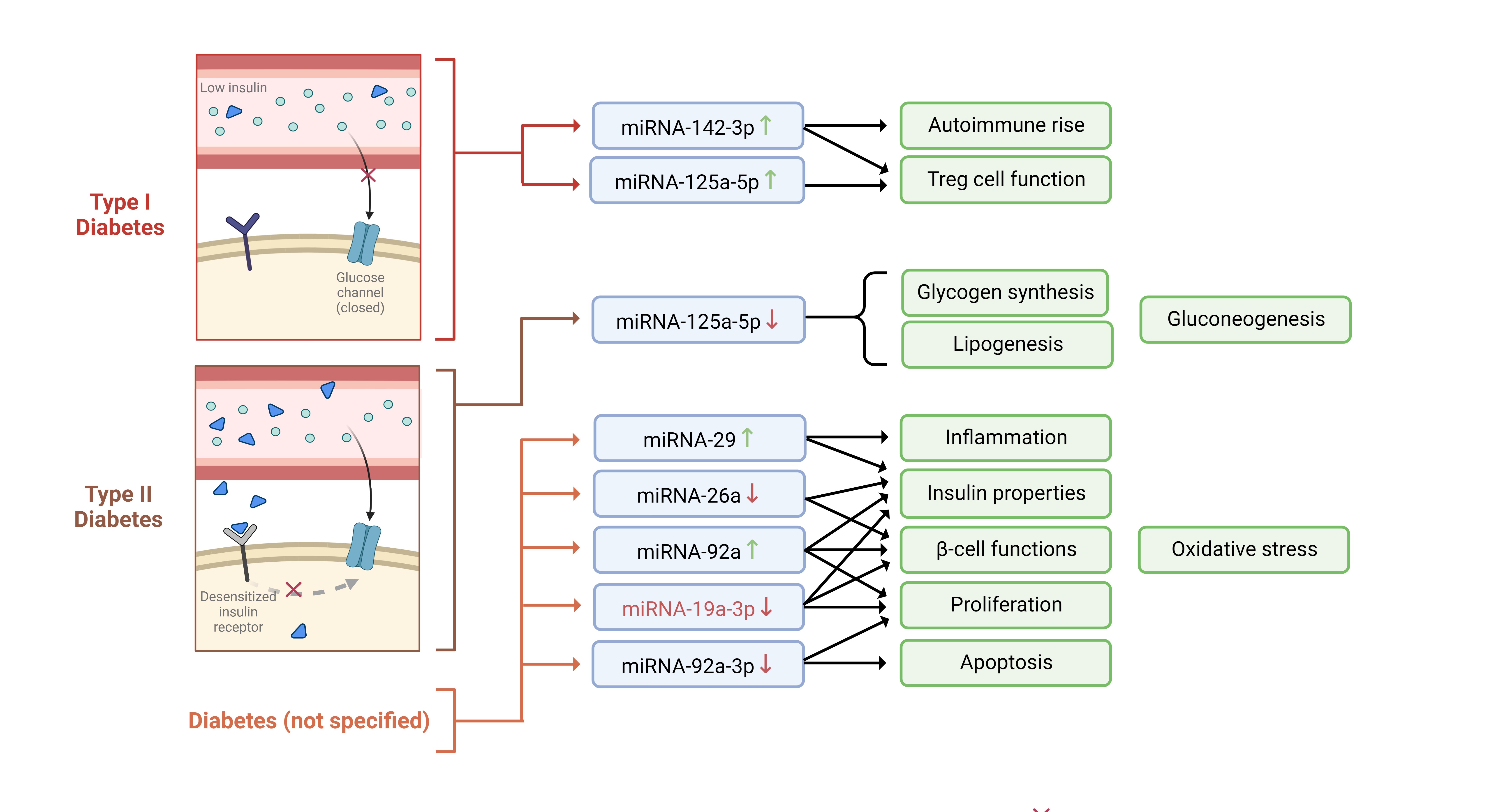

Supplement: Supplementary file 1 [file DataSheet1.ZIP › Figure R1.jpg]

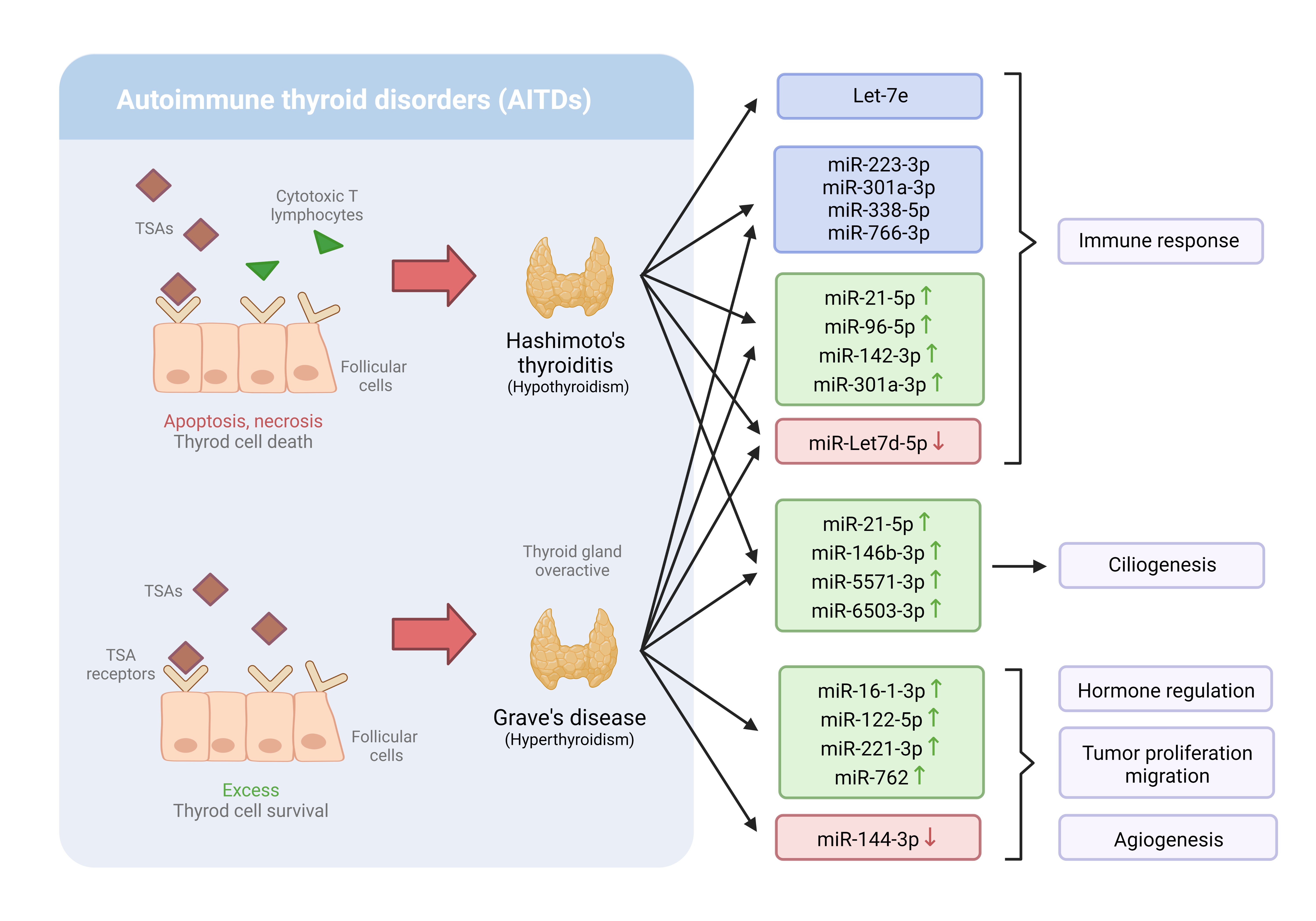

Supplement: Supplementary file 1 [file DataSheet1.ZIP › Figure R2.jpg]

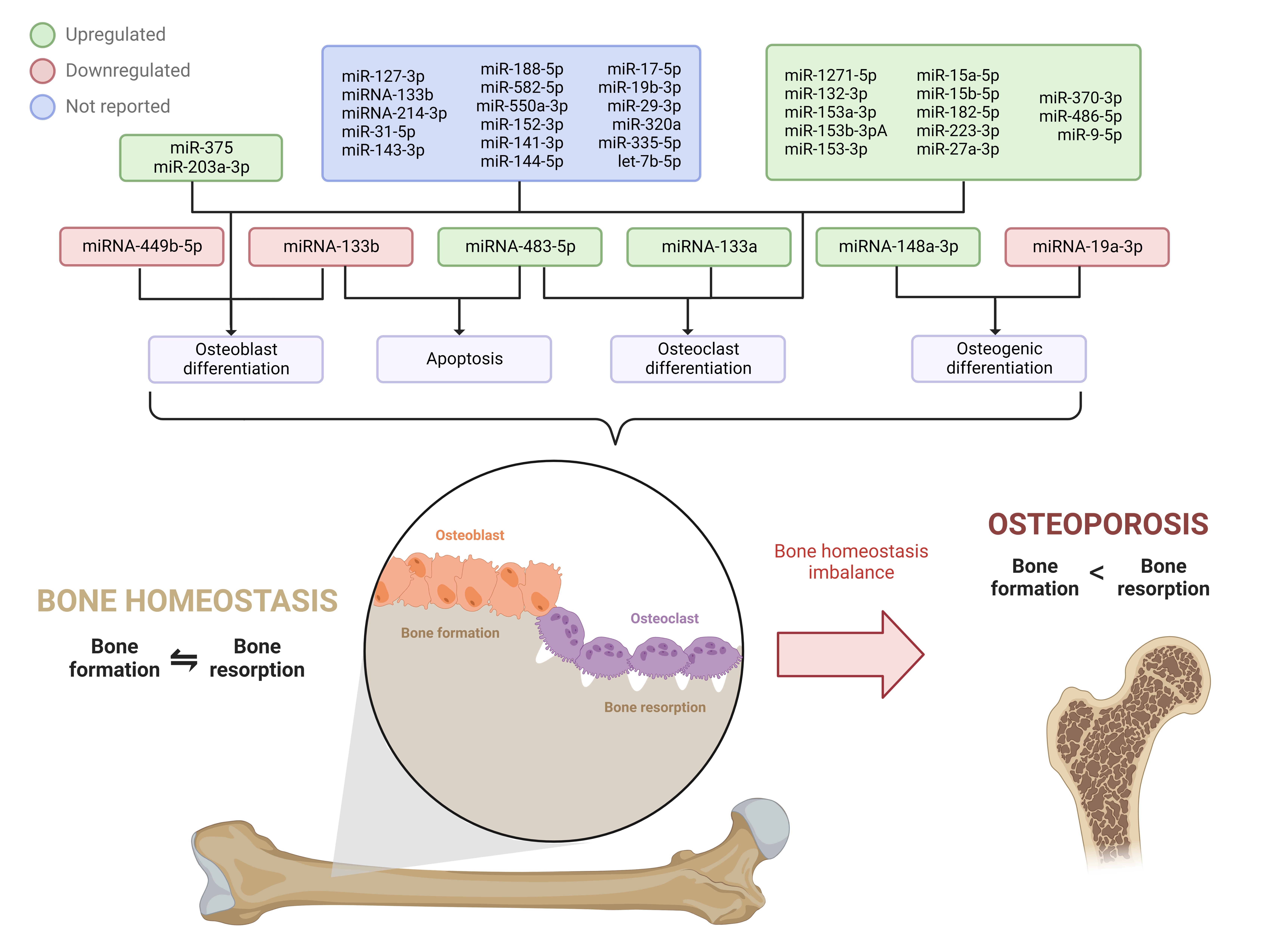

Supplement: Supplementary file 1 [file DataSheet1.ZIP › Figure R3.jpg]

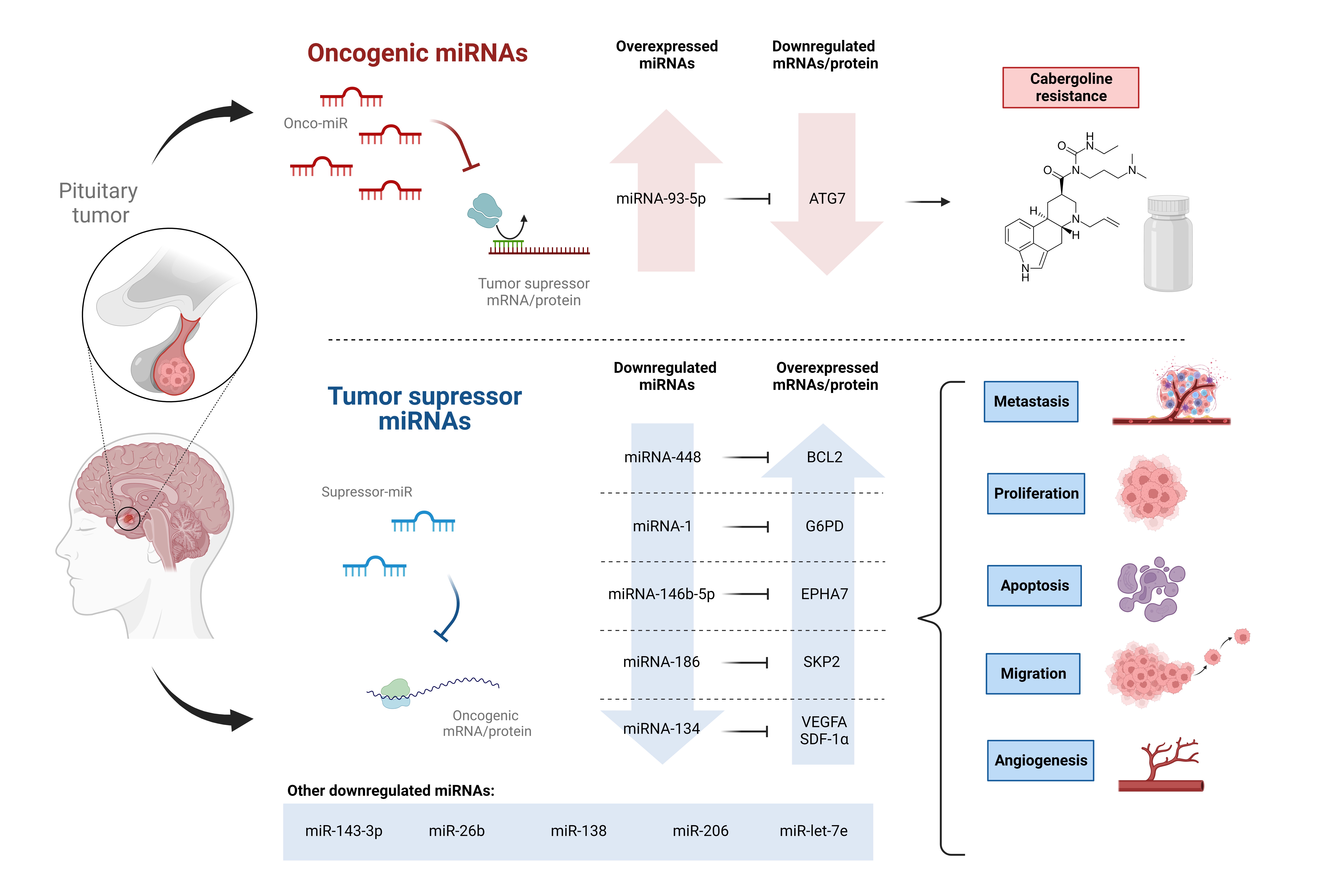

Supplement: Supplementary file 1 [file DataSheet1.ZIP › Figure R4.jpg]
